# Temporal changes in the viromes of Swedish Varroa-resistant and Varroa-susceptible honeybee populations

**DOI:** 10.1371/journal.pone.0206938

**Published:** 2018-12-06

**Authors:** Srinivas Thaduri, Barbara Locke, Fredrik Granberg, Joachim R. de Miranda

**Affiliations:** 1 Department of Ecology, Swedish University of Agricultural Sciences, Uppsala, Sweden; 2 Department of Biomedical Sciences and Veterinary Public Health, Swedish University of Agricultural Sciences, Uppsala, Sweden; University of Otago, NEW ZEALAND

## Abstract

The parasitic mite, *Varroa destructor*, in combination with the viruses it vectors, is the main cause for global colony losses of the European honeybee, *Apis mellifera*. However, an isolated honeybee population established in 1999 on the Island of Gotland, Sweden has naturally acquired resistance to the mite, and has survived without mite control treatment for more than 18 years. A recent study has shown that this mite resistant (MR) population also appears to be resistant to *Black queen cell virus* (BQCV) and *Sacbrood virus* (SBV) and tolerant to *Deformed wing virus* (DWV), relative to nearby mite susceptible (MS) honeybee populations. In this study, RNA sequencing was employed to corroborate these previous findings and identify other viral factors that may play a role in the enhanced survival of this mite resistant honeybee population. Two additional honeybee-infecting viruses, *Apis rhabdovirus-1* (ARV-1) and *Lake Sinai virus* (LSV), were identified and near-complete genomes of these two viruses were obtained. Phylogenetic analyses of the assembled virus sequences revealed consistent separation between the MR and MS honeybee populations, although it is unclear whether this is due to pre-existing differences between the viruses in the two populations when they were established, and isolated, or due to virus genetic adaptation towards reduced virulence in the MR population, to promote colony survival. Reverse transcription quantitative polymerase chain reaction(RT-qPCR) analyses show higher ARV and LSV titres in MS colonies compared to MR colonies, gradually increasing from summer to autumn 2009, and reaching maximum titres in the following spring 2010. While the DWV and BQCV titres in MR colonies increased between autumn 2009 and spring 2010, the SBV practically disappeared entirely by spring 2010. Possible explanations for the apparent virus tolerance or resistance in the Gotland mite-resistant honeybee population are discussed.

## Introduction

The Western honeybee (*A*. *mellifera*) is an economically important insect that plays a vital role in pollination of various agricultural crops [1]. Recent losses of managed honeybee colonies in the United States and in Europe have had a serious negative impact on the apicultural industry as well as on the agriculture-based food production industry due to the high demand on pollination services provided by honeybees [2]. Multiple stressors have been found to negatively influence bee health and lifespan [3] but it is clear that honeybee parasites and pathogens are the leading cause of colony death around the world [4]. Among all stressors, the ectoparasitic mite *Varroa destructor* is considered the major threat to honeybee health and the global apiculture industry [5]. Since shifting hosts from the Asian honeybee (*Apis cerana*), to the western honeybee (*A*. *mellifera*) in the mid-20^th^ century, the mite has spread around the world [6]. The mite feeds on the bee by injuring the cuticle of pupae and adults [7]. The honeybees then suffer due to the loss of hemolymph, reduced learning capability [8], host immunosuppression [9], and a reduced lifespan of adult bees [10,11]. However, the mite also acts as a vector for several honeybee RNA viruses, which are considered the major contributing factor to the worldwide collapse of honeybee colonies [12,13]. In particular, *Deformed wing virus* (DWV), considered a benign virus before the introduction of the mite, has become one of the most lethal honeybee pathogens with a global distribution due to its close association with the mite which acts as a vector of this virus [4]. Malformed wings, short and bloated abdomens and body miscolouring are typical symptoms related to the vectoral transmission of DWV by mites during the developmental stages of bee pupae [14,15]. In the absence of mites, DWV persists within the colony as a largely innocuous infection, through a combination of horizontal and vertical transmission routes [13,15–17]. As the mite population grows exponentially during the season, the increased virus transmission opportunities lead to overt infections and an excessive number of flightless, dysfunctional adult bees, which eventually results in the death of the colony in 2 to 3 years, unless active mite population control strategies are implemented [18,19]. For this reason, feral and wild honeybee colonies in Europe and North America have been nearly eradicated, due to the absolute necessity of active mite control for colony survival[20].

Despite these lethal effects of the mite on *A*. *mellifera*, certain subspecies, most notably in African (*A*.*m*. *scutellata*) and the African-derived bees in South America, appear to be resistant to Varroa and survive without mite control treatment by naturally maintaining low mite populations within colonies [20–22]. Moreover, although virus infections have been detected in Africa and African-derived honeybees, large colony losses due to mites and/or their associated/transmitted viruses are not observed [23–25]. This indicates that these populations might have developed a tolerance to certain pathogens in addition to their resistance against the mites [25].

There are few unique honeybee populations in North America and Europe that have been exposed to uncontrolled mite infestations and have survived without active mite control for more than 15 years [22,26–28]. Among these, the most comprehensively studied honeybee population is located on an isolated peninsula on the Island of Gotland, Sweden [22]. Studies have revealed that this population has naturally acquired mite resistance through minimal apicultural management and the deliberate absence of Varroa control [29–31]. The Gotland mite-resistant honeybee population exhibits a number of relevant colony level mite-resistant traits, such as small colony size [29] and reduced mite reproductive success, that together limit the mite population growth relative to mite susceptible colonies [30,31], leading to considerably lower mite infestation rates, especially towards the end of the season [32].

One of the more recent findings from this population was a tolerance to DWV, since colonies of the Gotland population survive with high DWV infection levels that are otherwise lethal to mite-susceptible honeybees [32]. Additionally, the Gotland mite-resistant honeybees appear to be resistant to other honeybee-infecting viruses such as *Black queen cell virus* (BQCV) and *Sacbrood virus* (SBV), which diminish by several orders of magnitude before the critical wintering phase of the honeybee life cycle [32], both in absolute terms and relative to nearby mite-susceptible honeybee populations. Although these viruses only cause symptomatic disease in larvae and are not usually lethal to *A*. *mellifera* at the colony level, they do affect adult bees, in particular, an aversion to pollen foraging and processing for SBV [33,34], and can therefore still be detrimental to general colony health and overwintering ability[35,36].

The objective of the current study is further analyze and extend the study by Locke et al. 2014 [32] by using a target-free metagenomic approach to identify possible additional viral factors that may be important to the enhanced survival of this unique population of mite-resistant honeybees.

## Materials and methods

### Sample origin

The samples derived from a seasonal survey in 2009–2010 of mite-resistant (MR) and mite-susceptible (MS) honeybee colonies on the island of Gotland, Sweden [32]. The mite-resistant colonies are part of an isolated honeybee population established in 1999 on Näsudden peninsula (N 57.072565 E 18.216705). The mite-susceptible colonies were located N 57.135736 E 18.312718, about 9.8 km from the mite-resistant population, *i*.*e*. well beyond the normal flight distances of worker bees, queens and drones [37] and were part of a self-contained local commercial beekeeping operation. The two populations were managed separately, with no exchange of bees between them. The MS population was under active Varroa management through drone removal and treatment with formic and/or oxalic acid as per Swedish recommendations until autumn 2007, after which they were managed identically to the MR population, *i*.*e*. without active Varroa or swarm management, but including the harvesting of surplus honey, and supplemental winter feeding as required [29]. The sample collection details can be found in Locke et al., 2014 [32]. In short, bulk samples of 30 adult bees were collected from the brood chambers of fourteen MR and twelve MS colonies at three different times during the late summer and autumn of 2009: July 28^th^, August 26^th^ and October 7^th^. The MS colonies all died during the winter 2009–2010. The eight surviving MR colonies were sampled again on May 17^th^, 2010. All samples were collected with the permission of the landowner and did not involve endangered or protected species. The samples were stored at -20°C [38] until total RNA was extracted from the bees as described previously [39] using the RNEasy manufacturer’s protocol for plant tissue (Qiagen). Eluted RNA was stored as two 25-μl aliquots at -80°C.

### Ion Torrent library preparation and RNA sequencing

The total RNA from all the individual MR or MS colonies at each sampling occasion was pooled in equimolar amounts and subjected to Poly (A) mRNA enrichment using the MicroPoly(A)Purist Kit (Ambion) according to the manufacturer's protocol, resulting in four MR and three MS population-level RNA samples: one each for July, August and October 2009 plus one MR sample for May 2010. The enriched mRNA samples were converted to Ion Torrent sequencing libraries and sequenced by the National Genomics Centre, SciLifeLab, Uppsala, Sweden. Briefly, the quality of the mRNA was checked using the Bioanalyzer RNA Pico chip (Agilent), following which the mRNA was fragmented with Ribonuclease III enzyme and the resulting fragments were ligated to adaptor sequences. cDNA synthesis, amplification and construction of RNA sequencing libraries was done using the Ion Total RNA-Seq v2 kit (Thermo Fisher Scientific). An Ion Torrent Personal Genome Machine (PGM) System was used for the sequencing of libraries using Ion 318 V2 chips and 400 bp read length chemistry.

### Bioinformatic analysis

The raw sequencing reads were trimmed to remove adaptor sequences and filtered to remove sequences with low quality scores (Q<20) with PRINSEQ version 0.20.4. The Kaiju web server was used for the metagenomics classification of reads, using the following setting: Greedy run mode with SEG filter, minimum match length = 11, minimum match score = 75, and allowed mismatches = 5 [40]. The Kaiju NCBI BLAST *nr +euk* database (103M protein sequences from Bacteria, Archaea, Viruses, Fungi and microbial eukaryotes, updated 2017-05-16) was selected as the reference database. The taxonomic assignments of the reads were visualized using Krona [41]. The genome sequences of known and novel viruses were assembled as follows: For each library separately, the host specific reads were removed by mapping to the *A*. *mellifera* genome assembly, Amel_4.5 using Bowtie2 [42]. The unmapped reads were extracted and assembled *de novo* by using Trinity [43]. The contigs thus generated were compared to the NCBI nucleotide and protein databases using BLASTn and BLASTx, respectively using an E-value cut-off of 1E-03. All contigs identifying with virus sequences were aligned to their respective virus database reference genome sequences using the CodonCode Aligner software 6.0.2 (CodonCode Corporation) and concatenated, in order to generate study-specific reference genomes for each virus. The individual reads from each sample were then re-mapped back to the various study-specific virus reference genomes using Bowtie2 and these mapped reads were extracted and counted using Samtools [44].

### Phylogenetic analysis

For each RNA sample, the mapped reads for each virus were collapsed into consensus sequences, with a >5x coverage threshold for inclusion of the character, so as to neutralize any bias from sequencing errors in the phylogenetic analyses. The sample-specific consensus genomes were aligned to each other and the full-length genomes of the major variants for each virus, using the CLUSTAL-W modality of MEGA6, with reduced penalties for gap inclusion and extension, so as to accommodate both relevant micro-variation and gaps in the alignments. Only full-length outgroup sequences were included in the alignments, explaining the absence of a number of LSV strains that have not yet been fully sequenced. The final alignments were checked for consistency and accuracy prior to inclusion in phylogenetic analyses. The evolutionary history for each virus was inferred using the Maximum Likelihood method based on the Tamura-Nei model[45] as implemented by MEGA6[46], with the trees with the highest log likelihood retained. The initial trees for the heuristic searches were obtained automatically by applying Neighbor-Joining and BioNJ algorithms to a matrix of pairwise distances estimated using the Maximum Composite Likelihood (MCL) approach, and then selecting the topology with superior log likelihood value. All positions containing gaps and missing data were excluded from the analyses. The percentage of trees in which the associated taxa clustered together was determined by bootstrap analyses involving 500 replicates. The numerical summary of the phylogenetic analyses and the accession numbers of the main virus reference sequences used are summarised in S2 Table.

### Completion of viral genome sequences

The sample-specific consensus sequences for the different viruses did not always cover the entire genome, and in some cases (SBV May-2010 and LSV July 2009) were missing entirely. The initial assembly of the study-specific BQCV sequences contained a ~1000 nt gap in the non-structural protein region, despite abundant coverage either side, implying that the relevant reads probably existed, but for some reason refused to assemble on the NCBI BQCV reference genome. The gap was therefore filled by Sanger sequencing PCR amplicons prepared from primers specific to the assembled BQCV sequence. The reads were then re-assembled onto this newly completed, study-specific BQCV sequence, where all the reads missing from the original gap were indeed recovered. Sanger sequencing of several PCR amplicons (S3 Fig) was also used to confirm the identity of the LSV and ARV-1 NGS sequence assemblies. In all cases, the Sanger sequencing matched 100% of the corresponding assembled sequences. Since the clearest separation between the virus sequences was according to the bee population of origin, the assembled sequence data from the individual samples was pooled by a population of origin (MR or MS) across the season to obtain the population-specific full-length consensus genome sequences for each of the viruses identified.

### cDNA synthesis and RT-qPCR analysis

The RNA from the 2010 MR samples was extracted at the same time as the 2009 samples, described previously[32]. For DWV, SBV and BQCV, the 2010 samples were assayed with the same One-Step RT-qPCR approach used previously [32], to maintain consistency within these datasets. However, because of the limited amount of RNA remaining from the 2009 samples, we had to use a Two-Step RT-qPCR approach for the LSV and ARV-1 assays. In our lab, there is minimal difference between the One-Step and Two-Step RT-qPCR approaches with respect to target quantification, and these differences are furthermore largely randomly distributed [38]. For the Two-Step RT-qPCR protocol, 400ng RNA from each sample was reverse transcribed in 20 μl volumes into cDNA, using random hexamer primers and the Invitrogen SuperScript-III first strand cDNA synthesis kit (ThermoFisher Scientific, Waltham, Massachusetts, USA) following manufacturer's instructions. The reaction conditions were: 25°C for 10 min, 37°C for 120 min, and 85°C for 5 min. The resulting cDNA was diluted 10-fold with sterile water and stored at -20°C. The abundance of LSV and ARV-1 cDNA in each sample, as well as that of the internal reference gene RP49 [39], was subsequently determined by RT-qPCR, using the EvaGreen qPCR kit (BioRad, Hercules, California, USA). The primers for the LSV and ARV-1 diagnostic RT-qPCR assays were designed in conserved areas of the respective assembled genomes, based on the aligned viral reads and contigs from the MR and MS samples. The primer design was optimized with respect to amplicon length, annealing temperature and codon redundancy [47] for maximum compatibility with the optimal qPCR cycling profile for the EvaGreen qPCR reagents. The LSV, ARV-1 and RP49 mRNA targets were amplified in 10 μl reaction volumes containing 2 μl diluted cDNA template and 0,3 μM of each primer, with the following cycling profile: 2 min at 95°C denaturation, followed by 35x[5 sec:95°C denaturation– 10 sec:58°C annealing, extension and data collection], followed by a Melting Curve analysis for determining the specificity of the amplification products, by incubating at 60 sec:95°C, 60 sec:65°C and fluorescence reading at 0.5°C increments between 65°C and 95°C. Included in each qPCR run was a ten-fold dilution series of known amounts of each target, for absolute quantification.

### Data conversion and normalization

The qPCR data were first screened for the presence of secondary RT-PCR products through visual inspection of the Melting Curve (MC) analyses. Only data where both duplicate assays gave a positive result by MC analysis were retained for quantitative analyses. The average Cq values were converted to Standard Quantity (SQ) values through the use of the external calibration curves established by the ten-fold dilution series for each target. These data were then multiplied by the various dilution factors throughout the methodology to estimate the copy number of each target per bee. These raw titres were then normalized to the average of their RP49 values, to correct for sample-specific differences in the quality and quantity of RNA [36]. These normalized titres were subsequently converted to log-scale, reflecting the natural distribution of virus titres in honeybee colonies [47]. Normalized viral read counts obtained from the RNA sequencing data were calculated by dividing the number of reads of each virus by the number of total reads and by the size of the respective virus reference genome (in kilobases), and then multiply this value by 10^6^ to generate the RPKM value (reads per kilobase of reference genome, per million mapped reads)[48].

### Statistical analysis

The differences between the MR and MS populations in the RT-qPCR derived viral titres were analysed using Welch's t-test, with P values less than 0.05 considered significant.

## Results

### Virome composition

**Fig 1:**
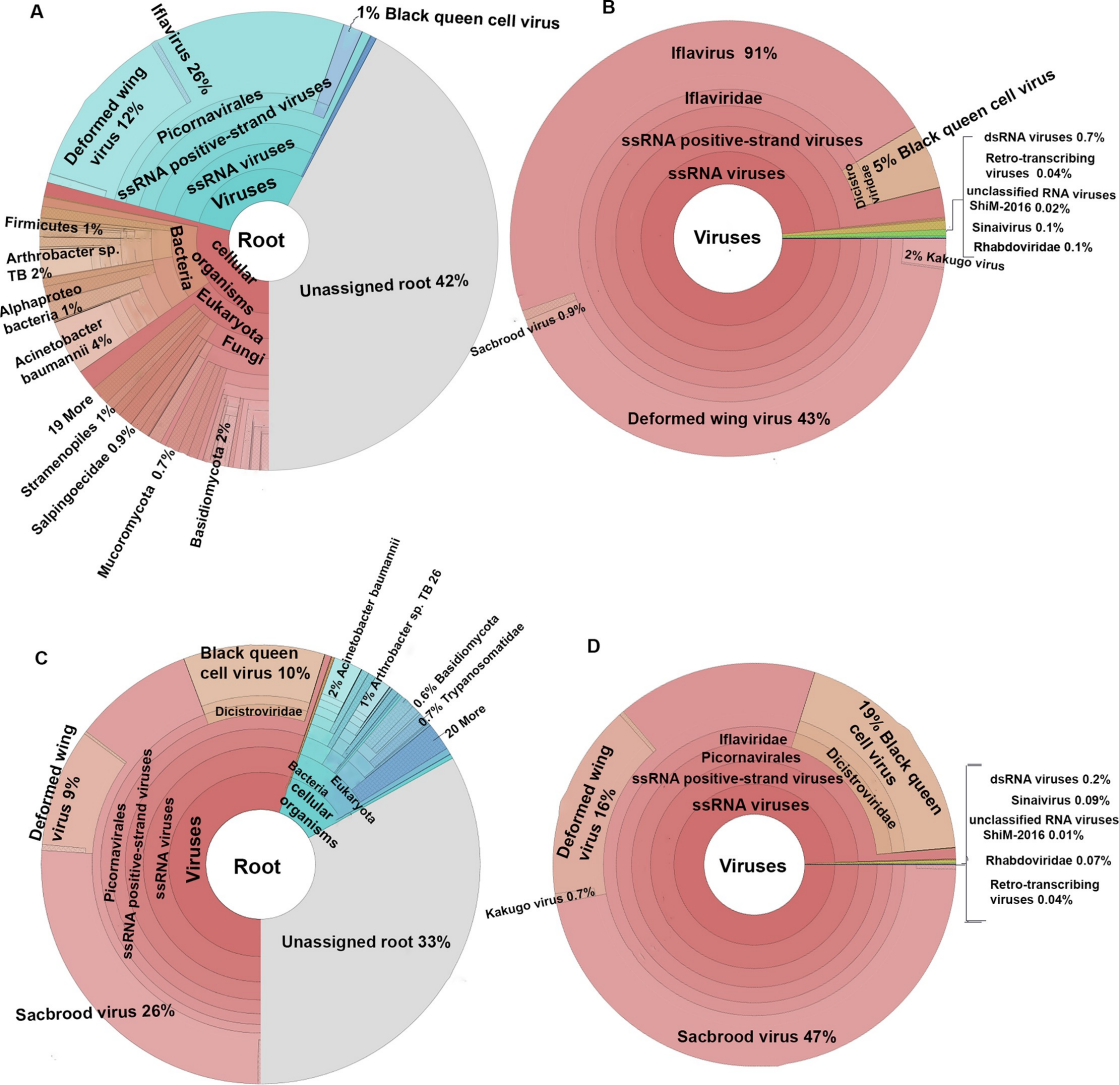
Microbial composition in MR and MS population. The distribution and classification of sequence reads for the October 2009 samples at the metagenomic root level (*i*.*e*. excluding all honeybee and other non-metagenome reads; A, C) and more specifically at the virome level (B, D) in the MR (A, B) and MS (C, D) honeybee populations, as visualized by Krona charts. The metagenomic classification was obtained through the Kaiju web server. The most abundant class of identified metagenome organisms is shown in brown-red colour (bacteria-fungi in A; viruses in B, C, D).

A total of 15501993, 24381209, 24713762, 24098024, 20820221 23036876 and 23469375 sequencing reads were obtained from the MR-Jul-09, MR-Aug-09, MR-Oct-09, MR-May-10, MS-Jul-09, MS-Aug-09, and MS-Oct-10 RNA samples respectively (S3 Table). The unassembled reads were filtered through the Kaiju Web server *nr +euk* database to extract all reads with a metagenomic character. The metagenome-related reads were classified as viruses or cellular organisms, with the cellular organisms in turn classified as either bacteria or eukaryotic organisms, mostly fungi. The majority of metagenome-classified reads in mite-resistant (MR) bees were assigned to cellular organisms (except for the July 2009 MR sample), whereas for mite-susceptible (MS) bees these were assigned to viruses (S3 Table). Fig 1 shows a visual representation of the distribution and classification of the metagenome-identified reads between different classes of microorganisms for the October 2009 samples. By this analysis, the most common virus for the MR population in October 2009 is DWV, while for the MS population this is SBV. Moreover, the proportion of viral reads in MS bees increased from July to October 2009, while the proportion of viral reads in MR bees remained relatively constant throughout 2009, except for July 2009. The proportion of viral reads in the MR colonies from spring 2010 that had survived the 2009–2010 winter was higher than during the summer and autumn of 2009.

Most of the viral reads in both populations were assigned to the three most common honeybee-infecting viruses in Scandinavia: Deformed wing virus (DWV), Sacbrood virus (SBV) and Black queen cell virus (BQCV). Two other known honeybee-infecting viruses: Lake Sinai Virus (LSV) and Apis rhabdovirus-1 (ARV-1), were also detected in both the MR and MS populations throughout the season. Several other known RNA viruses, mostly of plant origin, were also detected incidentally in the different libraries, e.g. Tomato black ring virus, Grapevine Anatolian ringspot virus and Beet ringspot virus. In addition, a minor number of viral sequence reads were ascribed to unclassified viruses with either retro-transcribing, single strand RNA or double strand RNA genomes (Fig 1).

### Full-length virus sequences

The full-length genome sequences of the major bee viruses in the MR and MS populations were assembled *de novo* from the sequence reads. The identity of the newly discovered Swedish LSV and ARV-1 isolates assembled from the IonTorrent sequence reads was confirmed through the Sanger sequencing of specific RT-PCR amplicons (S3 Fig), showing a 100% match with the assembled sequences. Iflavirus (DWV, SBV) and Dicistrovirus (BQCV) genomes are naturally poly-adenylated [49,50] and would be retained in the sequenced RNA samples after poly-A selection. Despite the fact that neither LSV [35] nor ARV-1 [51,52] are naturally polyadenylated, their genomes could be fully assembled from the IonTorrent sequencing output, including the non-transcribed regions. This most likely reflects the imperfection of enrichment procedures (*e*.*g*. poly-A selection), since other non-polyadenylated RNAs (*i*.*e*. from bacteria) were also recovered in abundance. This is not unusual: the original discovery and characterization of LSV was also from poly-A enriched RNA [53]. Moreover, since Rhabdoviruses express their genome through poly-adenylated mRNAs [54] the ARV-1 genome assembly may have been facilitated by enrichment for Rhabdovirus mRNA transcripts, if the virus was actively reproducing in these bees. Indirect evidence that this may indeed have been the case, and that ARV-1,therefore, replicates in bees [52], comes from the uneven distribution of positive-strand (mRNA plus replication intermediates) vs negative-strand (genomic RNA) reads across the assembled ARV-1 genome (S4 Fig), since IonTorrent RNA sequencing marks the orientation of the original RNA molecule, thus allowing positive and negative strand reads to be distinguished (Ion Total RNA-Seq v2 kit, Thermo Fisher Scientific). The relative overabundance of positive-strand (i.e. mRNA transcripts) reads is most noticeable towards the 5’ end of the genome, where the nucleocapsid proteins are located and thus a region that would naturally demand large amounts of mRNA transcript. The assembly also has considerably elevated coverage in that region, which is also consistent with the production of generous amounts of mRNA (S4 Fig). The status of LSV as a honeybee-infecting virus is much better established, both through quantitative analyses of replication intermediates and the identification of virus particles in honeybees [35].

### Phylogenetic relationships

**Fig 2:**
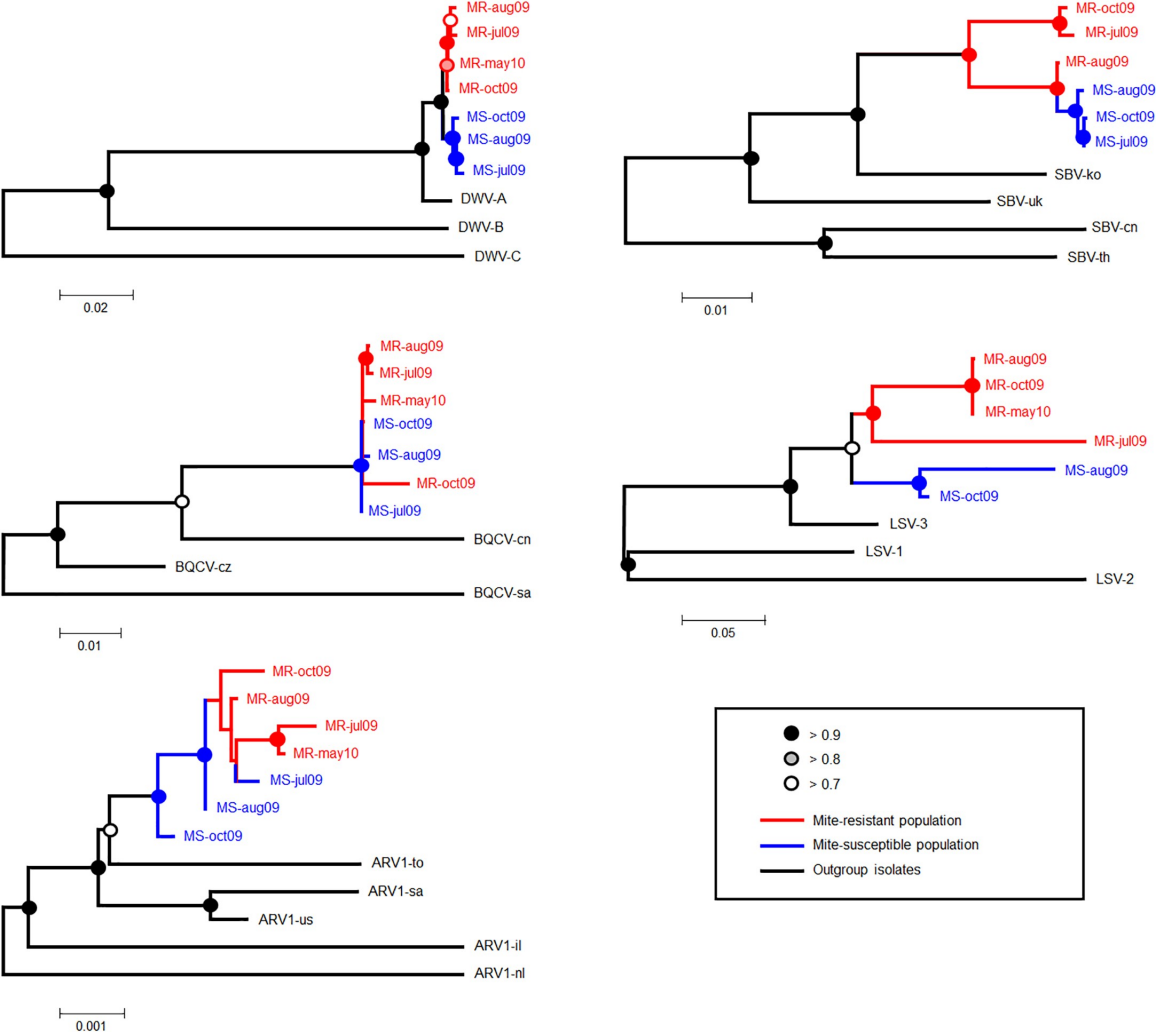
Phylogenetic analyses of major bee virus genome sequences. Reconstruction of the phylogenetic relationships between the consensus virus sequences in the different seasonal samples of the MR (red) and MS (blue) honeybee populations. The reconstructions are obtained through a Maximum Composite Likelihood (MCL) approach, with the most likely reconstruction presented. The open, shaded and closed circles indicate different probability intervals for the partition of the taxa across the node in question, based on 500 bootstrap replicates.

Phylogenetic analyses were conducted on the population-level consensus sequences of the principal bee viruses (DWV, SBV, BQCV, LSV and ARV-1) throughout the 2009–2010 season, obtained from the pooled seasonal samples of the MR and MS honeybee populations, involving 16 and 14 colonies respectively (Fig 2). For most viruses, these analyses show two levels of clustering: by honeybee population of origin (MR and MS), and separating both populations together from the outgroup taxa. However, within each population-specific cluster there is no consistent line-of-descent relationship between the different seasonal isolates. This pattern is clearest for DWV, SBV and LSV and less well resolved for BQCV and ARV-1. Only 25 SBV reads were recovered from the May 2010 MR sample, which was insufficient for assembling a consensus sequence. The other taxa that is missing is the July2009 LSV isolate of the MS population. This was because these samples contained mostly very short reads that were difficult to assemble on a genome that is naturally highly variable, resulting in large and ill-defined gaps in the multiple alignments. We therefore, chose to remove these taxa rather than attempt phylogenetic reconstruction on a dubious alignment. The DWV genomes found in the Swedish MR and MS populations clearly belonged to the DWV-A clade (Fig 2), which contains most of the DWV isolates identified to date (including Kakugo virus: Fig 1). Only a few reads were recovered matching the DWV-B clade (previously Varroa destructor virus-1; VDV-1), which is the other major DWV variant found world-wide. None were found for DWV-C, which thus far has only been identified in Great Britain [55]. Similarly, the MR and MS population SBV genomes clearly identify mostly with the European (SBV-uk) and Korean AmSBV-kor21 strains (SBV-ko) infecting *A*. *mellifera* [56] rather than the Chinese (SBV-cn) or Thai (SBV-th) strains, typically found only in *A*. *cerana*, but including at least one isolate from *A*. *mellifera* (AmSBV-kor19 from Korea[56]).

All currently known ARV-1 sequences, including the MR and MS population isolates, are >99% identical to each other. The LSV genomes, on the other hand, are extremely variable, both between the major strains and between the MR and MS population isolates of LSV3 (S2 Fig). The major difference between the MR and MS population isolates is a deletion of 8 amino acids in the MR sequence relative to the MS sequence (and the reference LSV3 sequence), located in the regions where ORF1 and the RdRp protein overlap. Immediately 5’ to this deletion is a variability hotspot of about 300 nucleotides which strongly affects the ORF1 amino sequence, but not the RdRp amino acid sequence. The second region of high nucleotide and amino acid variability between the MR and MS population LSV3 sequences is located in the N-terminal region of the capsid protein. This region is also variable between LSV strains, including large insertions/deletions that significantly affect the size of the capsid protein. There are a number of other, smaller insertion/deletions between the MR and MS population sequences, scattered throughout the genome.

### Virus titres

**Fig 3:**
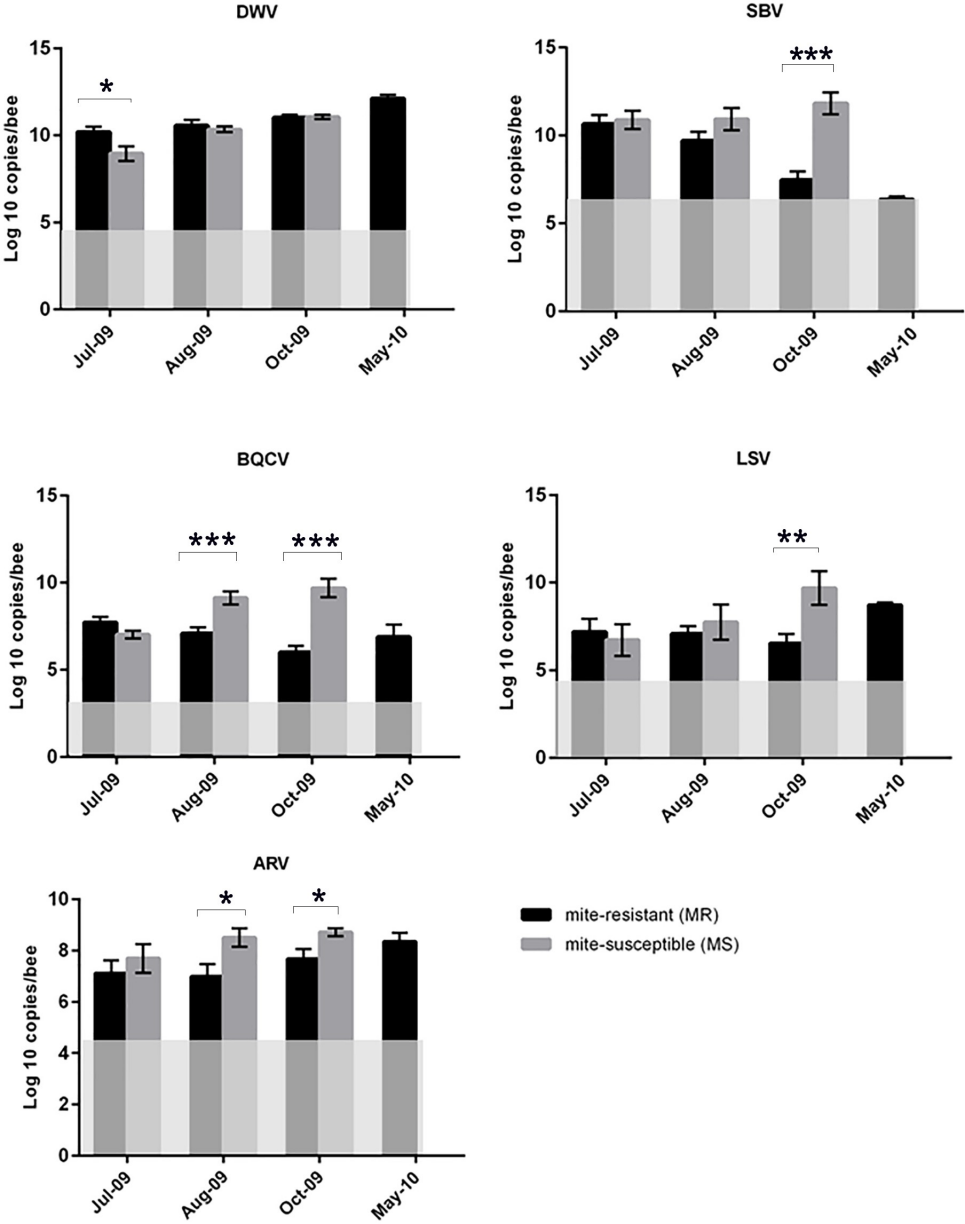
RT-qPCR data of major bee viruses. Virus titres in the 2009–2010 season for colonies in the MR and MS honeybee populations. Data are presented on a logarithmic scale for DWV, SBV, BQCV, LSV and ARV-1. Error bars indicate the standard deviation at each time-point. The opaque area in each graph (May-2010 for DWV, BQCV, SBV, and all seasons for LSV and ARV) represents the RT-qPCR detection threshold. The data for DWV, SBV, and BQCV for 2009 is from our previous study [32]. The corresponding data for 2010 and all data for LSV and ARV-1 is new. The asterisks indicate statistically significant differences, as determined by Welch’s t-test (* = P < 0.05, ** = P < 0.01, *** = P < 0.001).

The seasonal fluxes in the DWV, SBV and BQCV titres for the MR and MS populations for 2009 have been described in our previous study[32]. These showed that the titres of these viruses were relatively similar for MR and MS colonies during the summer, but diverged as the colonies moved into autumn and started producing winter bees, with the virus titres decreasing strongly in the MR colonies relative to the MS colonies. The corresponding May 2010 data for the surviving MR colonies is new. These show that between October 2009 and May 2010, the DWV and BQCV titres increased by several orders of magnitude, while SBV became almost undetectable in May 2010 (Fig 3 and S1 Fig). The patterns for LSV and ARV-1 most closely resemble those for BQCV, *i*.*e*. a strong decrease in the MR titres relative to the MS titres during the 2009 season, followed by a large increase in titre between October 2009 and May 2010 for the surviving MR colonies. The difference in virus titre between the MR and MS populations was insignificant in July 2009, for both LSV and ARV-1. The ARV-1 titres of the MS population increased between July 2009 and October 2009, while those of the MR population remained relatively constant, with a subsequent large and highly significant increase by May 2010. The differences between the MR and MS populations in ARV-1 titre were highly significant for both the August 2009 (*P* = 0.021) and October 2009 (*P* = 0.025) samples. The MS colonies had slightly lower LSV titres than MR colonies in July 2009, but these titres increased drastically in the MS population as the summer progressed to autumn, whilst in the MR population the LSV titres decreased. Again, in autumn 2009 the MR population had significantly (*P* = 0.01) lower LSV titres than the MS population. However, by spring 2010 the MR colonies had higher LSV titres than during the previous autumn.

## Discussion

The current study provides the first comprehensive analysis of viral-metagenomic landscape of the Swedish mite-resistant honeybee population, at a critical juncture during the honeybee season. Overall, there were no major qualitative differences in RNA metagenome composition between the MR and MS honeybee populations: the same type of viruses, bacteria and other parasites were recovered from both populations by the Kaiju server. Since only the RNA fraction was investigated here, the metagenome is logically dominated by viruses, which predominantly have RNA genomes. The non-viral RNA metagenomic sequences belonged primarily to a variety of bacteria (Firmicutes, Arthrobacter spp., Alphaproteobacteria, Acinetobacter spp.), fungi (Basidiomycetes, Mucoromycota) and a few higher Eukaryotic pathogens (trypanosomes and other unicellular flagellates). The fact that specific RNA molecules were recovered from these organisms suggests that they were replicating at some point in the bees. However, the RNA phase is inappropriate for analyzing the relative abundances of organisms with a DNA genome, since it reflects transcriptional activity rather than strict organism abundance, and therefore this information was not analysed further. Other methods are more suitable for analyzing the compositional abundance of these organisms [57]. Viruses occupy a significantly larger proportion of the metagenome for the MS population than for the MR population, which is confirmed by the RT-qPCR analyses. The vast majority of these viral reads belong to DWV, BQCV and SBV: well-known honeybee-infecting viruses in the *Iflaviridae* and *Dicistroviridae* families. A significant number of reads were also identified for two other honeybee-infecting viruses: Lake Sinai Virus (LSV), a positive-stranded RNA virus with indeterminate classification, and Apis rhabdovirus-1 (ARV-1) belonging to the *Rhabdoviridae* family. Since neither LSV nor ARV-1 has previously been described from Swedish bees, they were characterized here in greater detail.

Lake Sinai virus was first identified in a 10-month honeybee monitoring study carried out in the United States in 2009, where two main variants (LSV1 and LSV2) were described [53]. Since then, a large number of additional LSV variants have been discovered from surveys in the USA, Spain, Belgium, China and Australia [58–62][35], extending through LSV7, although most of these only through partial sequences. LSV strains have been detected in wild solitary bees of the *Megachilidae* and *Andrenidae* families [63] and in bumble bees [64], as well as in *A*. *mellifera* and Varroa [35]. Despite the high prevalence and global distribution of LSV, its pathology remains unknown. LSV infection has been linked to poor colony health [35] and Colony Collapse Disorder (CCD) [58], although causality has not yet been established. The LSV found in these Swedish colonies most closely resembles LSV3, although with only about 90% nucleotide identity to the reference genome, while the MR and MS population LSV3 isolates are only about 92% identical to each other, and include several larger and smaller deletions in both coding and non-coding regions. In this study, we observed higher LSV titres in MS colonies that subsequently died, than in corresponding MR colonies that survived. The extent to which these genetic and quantitative differences are significant for colony survival is unclear. The overall LSV titres throughout the survey are a couple of orders of magnitude lower than the titres of the main viruses (DWV and SBV) and LSV is naturally tolerant of very high levels of variability [35], of the type also seen between the MR and MS population isolates.

Apis rhabdovirus-1 (ARV-1) is an enveloped negative-sense single strand RNA virus that was recently discovered in honeybees and its varroa mites, and in the bumble bee *Bombus impatiens* [51,52]. A separate rhabdovirus, ARV2, was also described in these studies, as well as several other novel negative-stranded viruses. Negative-stranded viruses have also been identified in *Osmia cornuta*, a wild solitary bee [65]. ARV-1 has been detected in honeybee populations from North America, Europe, Middle East, Africa, and South Pacific, indicating that it has a near global distribution. Rhabdoviruses infect a broad range of species including plants, animals, and insects and are mostly transmitted by arthropod vectors[66]. Similar to LSV, the titres of ARV-1 in this study were consistently higher in the MS population than in the MR population, increasing steadily between summer and autumn 2009, and reaching maximum titre during spring 2010. Also the DWV and BQCV titres were much higher in May 2010 than at any time during 2009. The only virus to break this trend was SBV, which essentially disappeared from the MR colonies by May 2010, from a high during autumn 2009. The observation that all viruses investigated here show the same trend (increasingly lower relative and/or absolute titres in MR colonies as the colonies prepare for winter) suggests that there may be a common mechanism behind the phenomenon. In our previous study, we identified SBV as a possibly key virus for colony survival through its negative effects on protein processing by adult bees, which is of crucial importance to brood rearing and the production of vitellogenin, a key storage protein strongly linked to longevity [67].

The phylogenetic analyses consistently separate the MR and MS seasonal isolates by their population of origin: more clearly so for DWV, SBV and LSV, less obviously so for ARV-1 and BQCV. This separation into population-specific clusters could have a number of explanations. Although the MR and MS populations are geographically close, they have very distinct origins. The MS population is truly local while the MR population was established in isolation, from colonies collected throughout the Swedish mainland [37]. MR population is also closed, which means that there has been no exchange of microbiota with local colonies outside the area. The genetic differences between the MR and MS population viruses could therefore be pre-existing, from when the MR population was created in 1999 [37], or could be the result of the selection for survival in the mite-resistant population, perhaps resulting in reduced virus virulence, which would be expressed as increased tolerance or resistance of the MR population to virus infections [32].

The extent to which these observations on virus titres can explain the difference in colony survival between the two populations is limited. Many other parameters important for colony survival also differ drastically between the two populations, such as colony size [29] and mite reproductive success [30,31], making it difficult to gauge the relative importance of the decrease in virus titres for enhanced colony survival. More dedicated and precise experiments are needed to identify the mechanisms behind the reduced virus titres, particularly whether these reflect individual or colony level adaptations, and how those mechanisms in turn are related to increased colony-level survival.

## Supporting information

S1 Fig Major virus read-count data. Raw read-count data for the different viruses normalised to the total for each RNA sample. Data is given on logarithmic scale.
(TIF)

S2 Fig LSV genome map. Map of the genome organization for LSV1, LSV2 and LSV3, with the areas of major variability (downward arrows) and deletion (white block) between the MS and MR consensus LSV3 sequences indicated. The names of the different open reading frames (ORF) are shown.
(TIF)

S3 Fig Genome assembly maps. Schematic representation of the genomes DWV, SBV, BQCV, LSV and ARV-1, showing the location and direction of the contigs for the NGS assemblies separately for the MR (red) and MS (blue) honeybee populations. The green bars represent the RT-PCR products produced for sequence validation by Sanger sequencing. The tips of the brown arrows represent the location of the diagnostic RT-qPCR primers.
(TIF)

S4 Fig ARV-1 read map. Reads were mapped against the near full length study specific ARV-1 genome using the CodonCode aligner software 6.0.2. The read map illustrates the nucleotide coverage by viral genomic RNA (blue) and complementary RNA (red) reads.
(TIF)

S1 Table List of diagnostic RT-qPCR Assay primers and Sanger sequencing primers. Details of the diagnostic RT-qPCR assays used, including primer sequences, product size, linearity of the external calibration curves, Cq vs log_10_[target amount], over 6 orders of magnitude (r^2^) and the mean temperature of the main peak of the Melting Curve (T_m_).
(XLSX)

S2 Table Phylogenetic analyses and accessions. Supplementary data and information underlying the phylogenetic analyses, including the ML estimate of the optimum tree, the number of characters and taxa included in the reconstruction, as well as the accession numbers of the full-length consensus sequences for each of the viruses in the MR and MS honeybee populations (pooled across the seasons) and the most important outgroup sequences.
(XLSX)

S3 Table Basic information of the sequence data. Details about the number of IonTorrent sequence reads from the pooled seasonal samples of the MR and MS honeybee populations that map to either the honeybee genome, cellular microorganisms or viruses.
(XLSX)

S4 Table Statistical analysis of RT-qPCR data. Welch’s pairwise t-test analysis of the differences between the MR and MS honeybee populations in the DWV, SBV, BQCV, LSV and ARV-1 titres at different points in the 2009 season.
(XLSX)
