# Supplementary figures and images for: Temporal changes in the viromes of Swedish Varroa-resistant and Varroa-susceptible honeybee populations
Source: PLoS One. 2018 Dec 6;13(12):e0206938. doi: 10.1371/journal.pone.0206938 (PMC6283545; doi:10.1371/journal.pone.0206938)

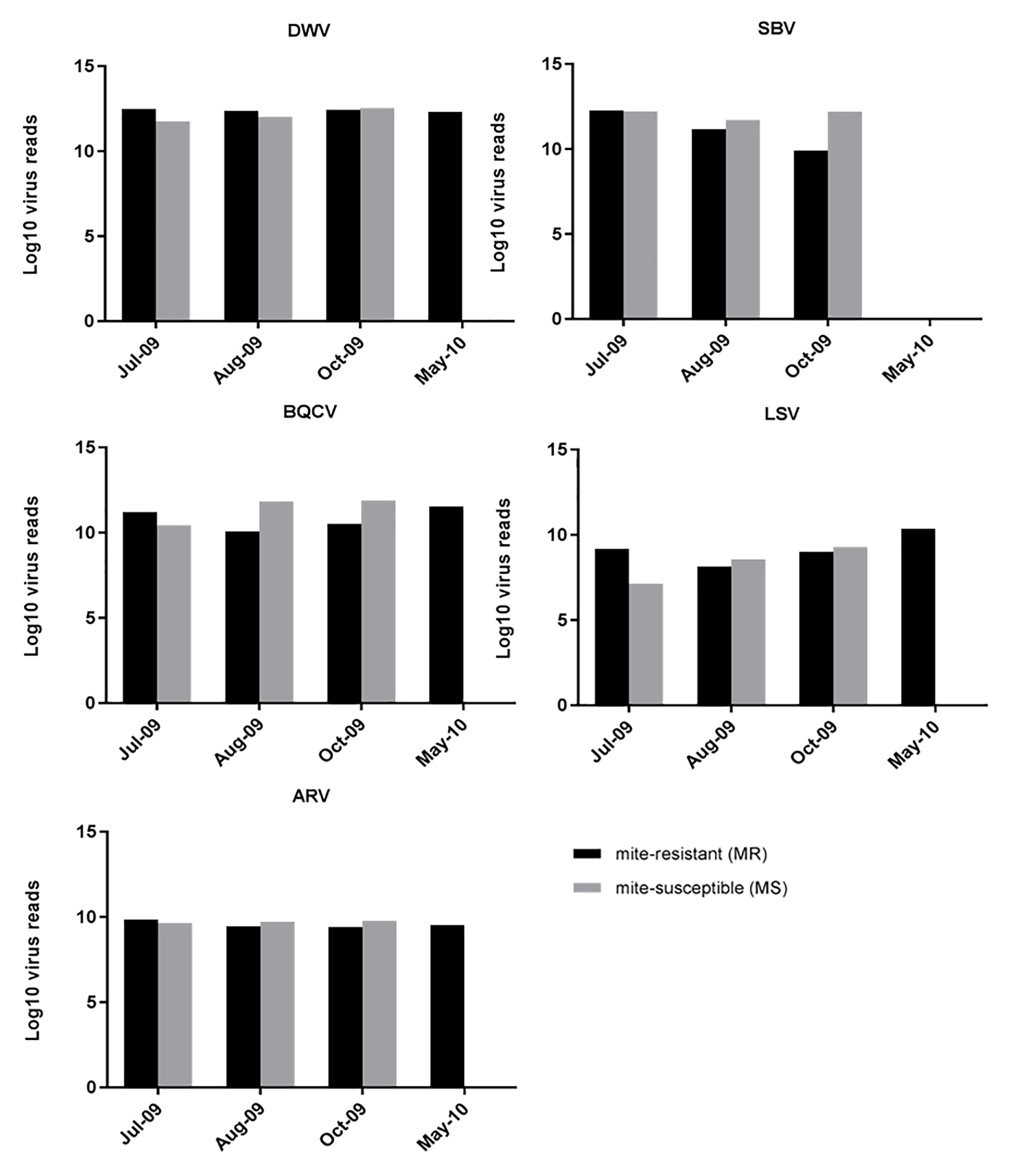

Supplement: S1 Fig — Raw read-count data for the different viruses normalised to the total for each RNA sample. Data is given on logarithmic scale. (TIF) [file pone.0206938.s001.tif]

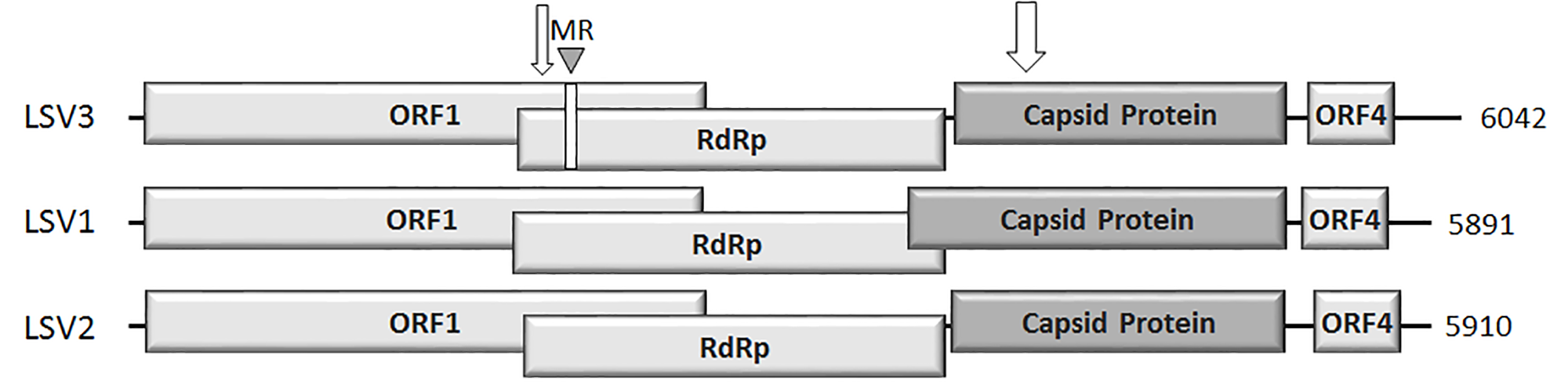

Supplement: S2 Fig — Map of the genome organization for LSV1, LSV2 and LSV3, with the areas of major variability (downward arrows) and deletion (white block) between the MS and MR consensus LSV3 sequences indicated. The names of the different open reading frames (ORF) are shown. (TIF) [file pone.0206938.s002.tif]

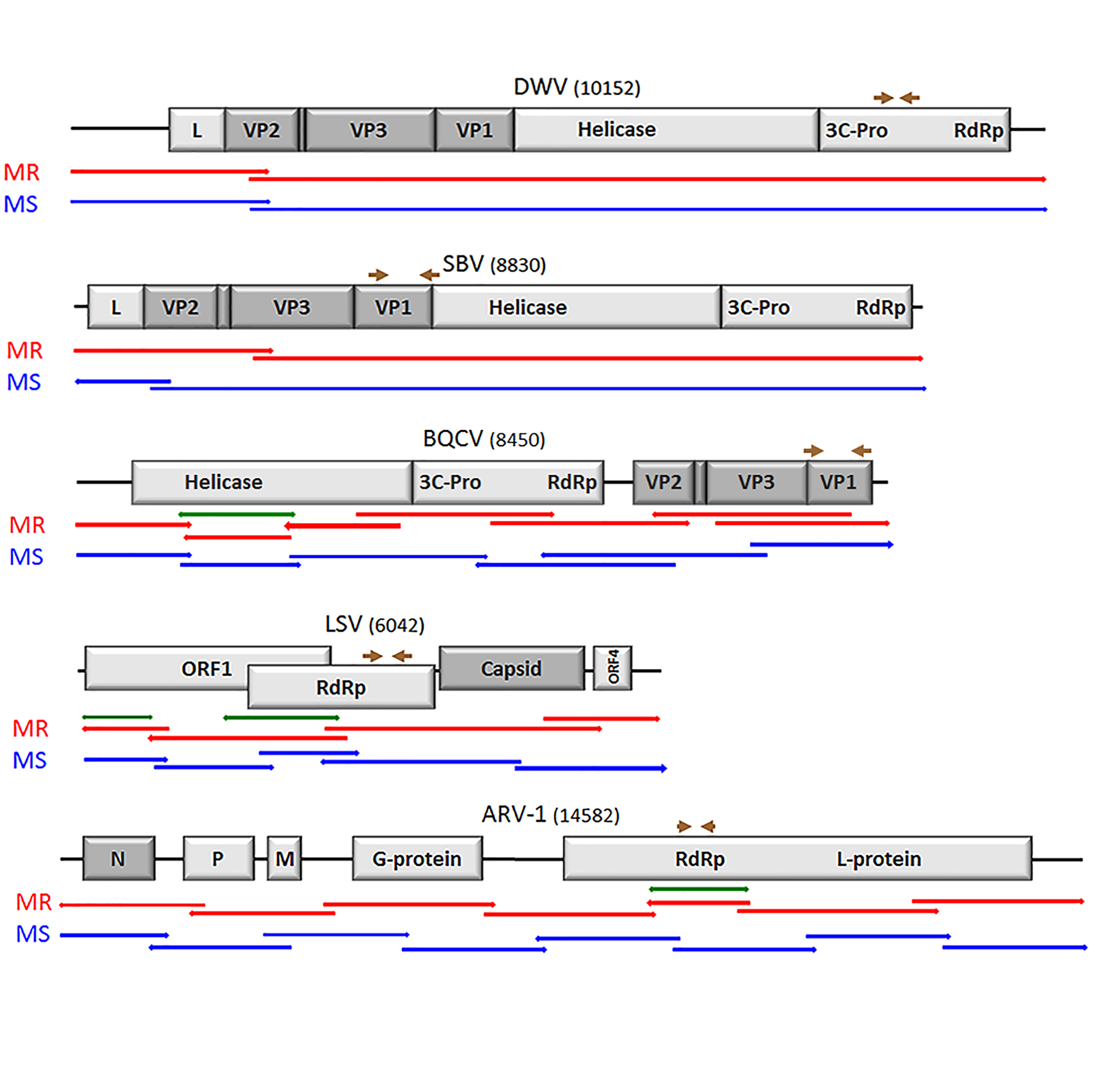

Supplement: S3 Fig — Schematic representation of the genomes DWV, SBV, BQCV, LSV and ARV-1, showing the location and direction of the contigs for the NGS assemblies separately for the MR (red) and MS (blue) honeybee populations. The green bars represent the RT-PCR products produced for sequence validation by Sanger sequencing. The tips of the brown arrows represent the location of the diagnostic RT-qPCR primers. (TIF) [file pone.0206938.s003.tif]

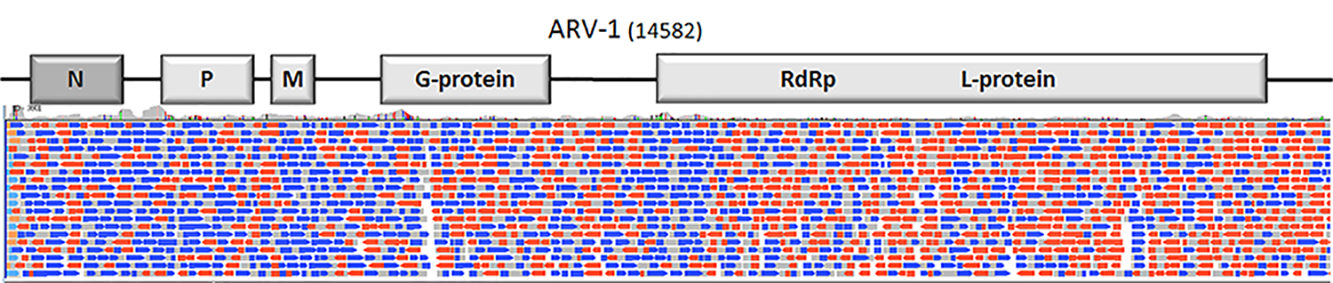

Supplement: S4 Fig — Reads were mapped against the near full length study specific ARV-1 genome using the CodonCode aligner software 6.0.2. The read map illustrates the nucleotide coverage by viral genomic RNA (blue) and complementary RNA (red) reads. (TIF) [file pone.0206938.s004.tif]
